# Mapping trends and hotspot regarding gastrointestinal microbiome and neuroscience: A bibliometric analysis of global research (2002–2022)

**DOI:** 10.3389/fnins.2022.1048565

**Published:** 2022-11-17

**Authors:** Jingjing Yang, Yihui Deng, Yuzhe Cai, Yixuan Liu, Lanyu Peng, Zheng Luo, Dingxiang Li

**Affiliations:** ^1^Key Laboratory of Hunan Province for Integrated Traditional Chinese and Western Medicine on Prevention and Treatment of Cardio-Cerebral Diseases, Hunan University of Chinese Medicine, Changsha, China; ^2^Hunan University of Chinese Medicine, Changsha, China

**Keywords:** gastrointestinal microbiome, neuroscience, bibliometric, visualization analysis, WoSCC, research trends, hotspots

## Abstract

**Background:**

Scholars have long understood that gastrointestinal microorganisms are intimately related to human disorders. The literature on research involving the gut microbiome and neuroscience is emerging. This study exposed the connections between gut microbiota and neuroscience methodically and intuitively using bibliometrics and visualization. This study’s objectives were to summarize the knowledge structure and identify emerging trends and potential hotspots in this field.

**Materials and methods:**

On October 18, 2022, a literature search was conducted utilizing the Web of Science Core Collection (WoSCC) database for studies on gut microbiota and neuroscience studies from 2002 to 2022 (August 20, 2022). VOSviewer and CiteSpace V software was used to conduct the bibliometrics and visualization analysis.

**Results:**

From 2002 to 2022 (August 20, 2022), 2,275 publications in the WoSCC database satisfied the criteria. The annual volume of publications has rapidly emerged in recent years (2016–2022). The most productive nation (*n* = 732, 32.18%) and the hub of inter-country cooperation (links: 38) were the United States. University College Cork had the most research papers published in this area, followed by McMaster University and Harvard Medical School. Cryan JF, Dinan TG, and Clarke G were key researchers with considerable academic influence. The journals with the most publications are “Neurogastroenterology and Motility” and “Brain Behavior and Immunity.” The most cited article and co-cited reference was Cryan JF’s 2012 article on the impact of gut microbiota on the brain and behavior. The current research hotspot includes gastrointestinal microbiome, inflammation, gut-brain axis, Parkinson’s disease (PD), and Alzheimer’s disease (AD). The research focus would be on the “gastrointestinal microbiome, inflammation: a link between obesity, insulin resistance, and cognition” and “the role of two important theories of the gut-brain axis and microbial-gut-brain axis in diseases.” Burst detection analysis showed that schizophrenia, pathology, and psychiatric disorder may continue to be the research frontiers.

**Conclusion:**

Research on “gastrointestinal microbiome, inflammation: a link between obesity, insulin resistance, and cognition” and “the role of two important theories of the gut-brain axis and microbial-gut-brain axis in diseases” will continue to be the hotspot. Schizophrenia and psychiatric disorder will be the key research diseases in the field of gut microbiota and neuroscience, and pathology is the key research content, which is worthy of scholars’ attention.

## Introduction

Gut microbiota represents a complex and dynamic microbial community in the gastrointestinal tract (Quercia et al., 2014). Since the dawn of humanity, these microbes have developed niche ecosystems tailored to particular habitats and adapted to the physiological requirements of their hosts (Lloyd-Price et al., 2016). The term “neuroscience” refers to studies in non-clinical fields like neurobiology and neurochemistry as well as clinical specialties like neurology, neurosurgery, neuropsychiatry, and psychology (Adolphs, 2015). The brain and gastrointestinal tract communicate in both directions through the hypothalamus-pituitary-adrenal (HPA) and microbiota-gut-brain (MGB) axes (Cenit et al., 2017; Wiley et al., 2017). We now understand that certain neurological and neuropsychiatric conditions, including Alzheimer’s disease (AD), Parkinson’s disease (PD), depression and anxiety, and autism spectrum disorder (ASD), may be significantly influenced by the gastrointestinal microbiome. This influence is also bidirectional, as shown by the comorbidities of certain neural pathologies and intestinal dysbiosis [such as irritable bowel syndrome (IBS), insulin-resistance, inflammatory bowel disease, or alterations in the ratio of bacterial species of the microbiota itself detected in patients with depression) (Wu, 2012). Physiologically, microbiota affects amygdala maturation in mammals, and baseline neuronal activity in the amygdala is altered in germ-free animals, leading to neurodevelopmental disorders (Stilling et al., 2015). Among the factors associated with the field of neuroscience pathology, the gut microbiota can affect a broad range of host neuroscience disorders by interacting with the host through immune, metabolic, neural, and endocrine pathways (Afzal et al., 2020). In recent decades, the relationships between the gastrointestinal microbiome and neuroscience in animals and humans have been widely studied. For instance, many probiotic strains known as “psychobiotics,” such as Bifidobacteria, Lactobacillus helveticus had a positive impact on cognition (Savignac et al., 2015; Ni et al., 2019), Saccharomyces boulardii (Tao et al., 2021) and Lactobacillus rhamnosus (Chivero et al., 2021), as well as multi-species probiotics, have been shown to improve neurological diseases by regulating intestinal flora (Ma T. et al., 2021; Rajanala et al., 2021). Multiple studies have presented evidence that nervous system disease leads to microbiome changes or microbiota changes lead to neuroinflammation that can trigger neurological diseases and indicate a causal relationship between them (Yang et al., 2020; Farooq et al., 2022). Many human studies have also shown that gut microbiota is closely related to the development of neurological diseases. Such as specific bacterial taxa commonly associated with mental disorders, including lower levels of bacterial genera that produce short-chain fatty acids (e.g., butyrate), higher levels of lactic acid-producing bacteria, and higher levels of bacteria associated with glutamate and GABA metabolism (Anonymous, 2022). Additionally, some clinical trials have been conducted to explore the feasibility of probiotics for the treatment of neurological diseases. Such as probiotics can improve cognitive function in patients with Alzheimer’s disease and major depression (Agahi et al., 2018; Rudzki et al., 2019).

The bibliometric analysis is one method for measuring the development of an area of study (Flay, 1986; Milat et al., 2011), and it may be applied to explore, analyze, and summarize the formation of knowledge’s structure in the spatiotemporal dimensions. This approach, which has been used in a variety of domestic and international fields, offers a diverse perspective that traditional literature reviews and systematic reviews cannot (Chen et al., 2014; Cesarano et al., 2021; Ma S. et al., 2021; Pan et al., 2021; Pasin and Pasin, 2021; Shi et al., 2021; Wu et al., 2021a; Fu et al., 2022). Bibliometric analysis has been widely used in the field of microbial science, such as microbiome and COVID-19 (Xavier-Santos et al., 2022), gut microbiota and host immune response (Ni et al., 2022), gut microbiota and atherosclerosis (Wang Y. et al., 2022), microbiome-gut-brain axis (Wang H. et al., 2022), gut microbiota and heart failure (Mu et al., 2022), gut microbiome and cancer (Zyoud et al., 2022), the intestinal microbiome and inflammatory bowel disease (Xu et al., 2022), gut microbiota and Parkinson’s disease (Cabanillas-Lazo et al., 2022), and gut microbiota and depression (Zhu et al., 2021).

The link between gut microbiota and neuroscience has not yet been subjected to a bibliometric study. However, the volume of research on gut microbiota and neuroscience has exploded, making it challenging to uncover specific data and make more accurate predictions. Therefore, bibliometrics and visualization were employed in this study to intuitively and methodically disclose the connections between gut microbiota and neuroscience, the objectives were to summarize the knowledge structure and identify emerging trends and potential hotspots in this field.

## Materials and methods

### Data collection

This study utilized the Web of Science Core Collection (WoSCC) database as its data source. The WoSCC database, one of the most comprehensive, systematic, and authoritative databases, has been extensively used by a substantial number of academics for scientometric analysis and visualization of scientific literature (Chen et al., 2020; Devos and Menard, 2020; Wu et al., 2021b; Zhang et al., 2021).

‘‘Gastrointestinal microbiomes’’ was searched in the Medical subject headings (MeSH) of PubMed to obtain Gastrointestinal microbiome-related terms. Then these terms were used to do searches on WoSCC, the search strategy was as follows: ‘‘Topic: [(gastrointestinal microbiomes) OR (microbiome, gastrointestinal) OR (gut microbiome) OR (gut microbiomes) OR (microbiome, gut) OR (gut microbiota) OR (gut microbiotas) OR (microbiota, gut) OR (gastrointestinal flora) OR (flora, gastrointestinal) OR (gut flora) OR (flora, gut) OR (gastrointestinal microbiota) OR (gastrointestinal microbiotas) OR (microbiota, gastrointestinal) OR (gastrointestinal microbial community) OR (gastrointestinal microbial communities) OR (microbial community, gastrointestinal) OR (gastrointestinal microflora) OR (microflora, gastrointestinal) OR (gastric microbiome) OR (microbiome, gastric) OR (gastric microbiomes) OR (intestinal microbiome) OR (intestinal microbiomes) OR (microbiome, intestinal) OR (intestinal microbiota) OR (intestinal microbiotas) OR (intestinal microflora) OR (microflora, intestinal) OR (intestinal flora) OR (flora, intestinal) OR (enteric bacteria) OR (bacteria, enteric)] AND Language: (English) AND Document Type:(Article OR Review) AND Web of Science Categories: (Neurosciences) AND Publication Date: (2002-01-01 to 2022-08-20)’’ (Searchable link)^1^. “Full Record and Cited References” of the catalogue are exported in “Plain Text” format and were named “download *.txt.” The search was completed on 18 October 2022, a total of 2,275 articles were chosen and then used to perform a bibliometric analysis.

### Data analysis and visualization

Excel 2016 was used to import and analyze data related to the number of articles published each year on the gastrointestinal microbiome and neuroscience. Use “thesaurus_terms.txt” in VOSviewer 1.6.17 to create thesaurus files, such as merged “ischemic-stroke” and “ischemic stroke,” “gut microbiome” and “gastrointestinal microbiomes,” “gut-brain-axis” and “gut-brain axis,” “Chinese acad sci” and “univ Chinese acad sci” in term analysis. We utilized VOSviewer 1.6.17 to locate the top prolific journals, institutions, countries/regions, citations of documents, co-cited references, terms, and related knowledge maps.

CiteSpace V (Version 6.1 R2)^2^ was used to detect a citation-burst analysis of WoS-generated keywords. Parameters of CiteSpace were set as follows: time slicing (2002–2022), years per slice (1 year), term source (keywords), node type (keywords), selection criteria (top 50), burstness (γ 1.0).

## Results

### Annual growth trend

**FIGURE 1:**
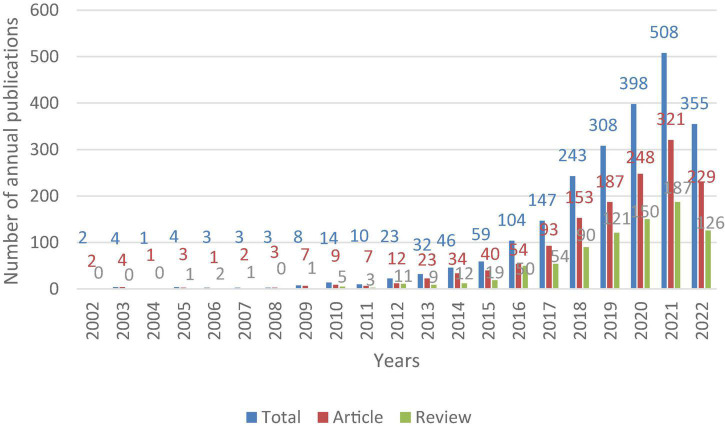
Trends in the number of publications for research in the gastrointestinal microbiome and neuroscience from 2002 to 2022.

There were 2,275 articles on the gastrointestinal microbiome and neuroscience in the WOSCC from 2002 to 2022 (August 20, 2022), including 1,433 original research articles and 842 reviews. The annual publication is exhibited in Figure 1. The growth of the publications showed three stages. The first stage was from 2002 to 2009, the second stage was from 2010 to 2015, and the third stage was from 2016 to 2022. The overall trend of articles and reviews was consistent with annual publications, but the number of articles was higher than that of reviews.

### Top active countries/Regions

**FIGURE 2:**
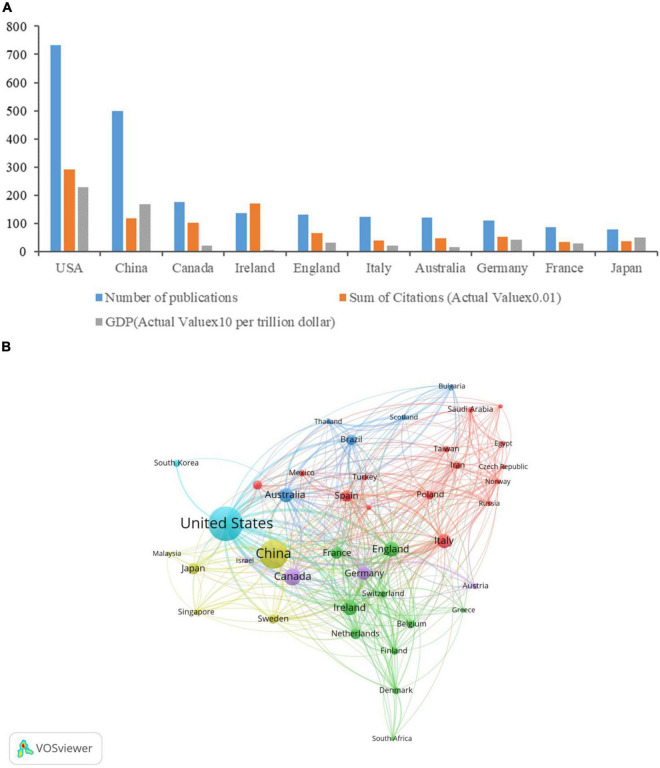
Contributions of different countries/regions to research on gastrointestinal microbiome and neuroscience. **(A)** Number of publications, sum of citations (Actual Value × 0.01), and GDP (Actual Value × 10 per trillion dollar) in the top 10 active countries/regions. **(B)** Cooperation network of countries/regions to study the gastrointestinal microbiome and neuroscience. Map of countries/regions cooperation showed 41 countries/regions with a minimum of 10 publications.

A total of 2,618 institutions from 83 countries/regions contributed to 2,275 publications. The United States produced the most publications (732, 32.18%), then China (500, 21.98%), Canada (177, 7.78%), Ireland (137, 6.02%), and England (132, 5.80%) (Table 1). Figure 2A shows that the number of publications in a country is positively correlated with its gross domestic product (GDP). Figure 2B is a cooperation network of countries/regions to study the gastrointestinal microbiome and neuroscience. This map showed 41 countries/regions with a minimum of 10 publications. The most cooperation with other countries/regions is the United States (links: 38), followed by England (links: 35), Australia (links: 32), Germany (links: 31), and Canada (links: 31).

**TABLE 1:** The top 10 countries/Regions to study the gastrointestinal microbiome and neuroscience.

Rank	Country	Counts	%	Citations	Avg. citations
1	USA	732	32.18%	29178	39.86
2	China	500	21.98%	11856	23.71
3	Canada	177	7.78%	10303	58.21
4	Ireland	137	6.02%	17035	124.34
5	England	132	5.80%	6631	50.23
6	Italy	124	5.45%	4011	32.35
7	Australia	122	5.36%	4754	38.97
8	Germany	111	4.88%	5187	46.73
9	France	86	3.78%	3548	41.26
10	Japan	80	3.52%	3657	45.71

### Top active institutions

**FIGURE 3:**
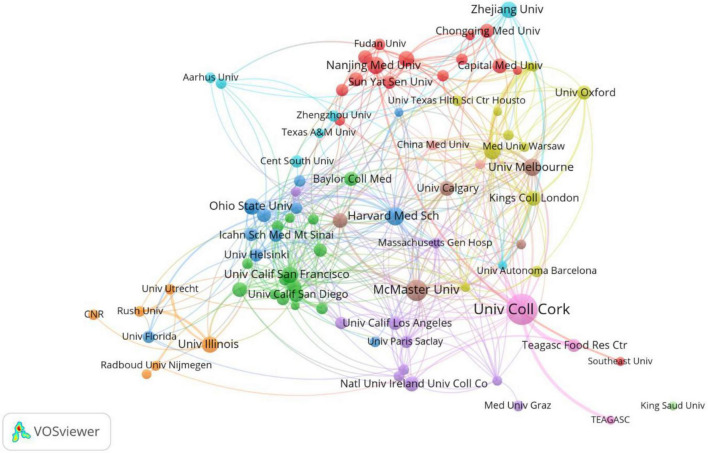
Cooperation network of institutions to study the gastrointestinal microbiome and neuroscience. Map of institutions’ cooperation showed 87 institutions with a minimum of 10 publications.

The top 10 institutions were from the USA (4/10), Australia (2/10), China (2/10), Ireland (1/10), and Canada (1/10) (Table 2). University College Cork (105, 4.62%) published the most papers, followed by McMaster University (50, 2.20%), Harvard Medical School (35, 1.54%), Nanjing Medical University (31, 1.36%), and the University of Melbourne (31, 1.36%) (Table 2). A collaboration network of institutions to research the gastrointestinal microbiome and neuroscience is shown in Figure 3. This map shows 87 institutions with a minimum of 10 publications. The Harvard Medical School has the most collaborations with other institutions (links: 31), followed by University College Cork (links:30), Deakin University (links:25), Massachusetts General Hospital (links: 21), and McMaster University (links: 21).

**TABLE 2:** The top 10 institutions research the gastrointestinal microbiome and neuroscience.

Rank	Institutions	Countries	Counts	%	Citations	Avg. citations
1	University College Cork	Ireland	105	4.62%	8093	77.08
2	McMaster University	Canada	50	2.20%	5165	103.30
3	Harvard Medical School	USA	35	1.54%	1447	41.34
4	Nanjing Medical University	China	31	1.36%	165	5.32
5	The University of Melbourne	Australia	31	1.36%	969	31.26
6	The Ohio State University	USA	30	1.32%	1655	55.17
7	Zhejiang University	China	30	1.32%	1848	61.60
8	University of California, San Francisco	USA	29	1.27%	1009	34.79
9	Deakin University	Australia	28	1.23%	728	26.00
10	University of Illinois	USA	28	1.23%	969	34.61

### Authors and co-cited authors

**FIGURE 4:**
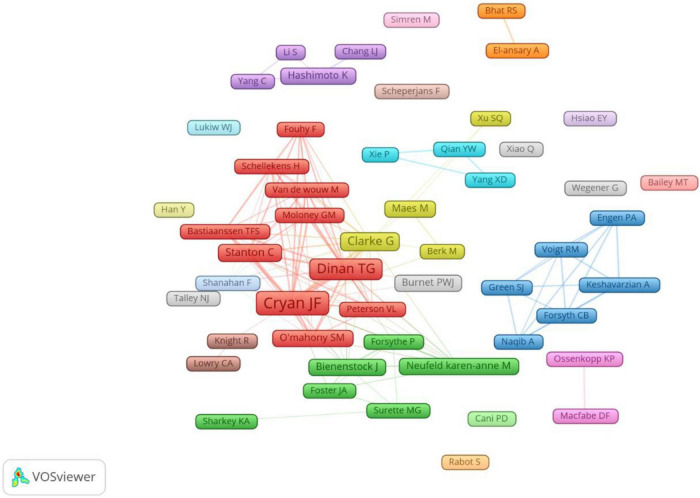
The co-occurrence map of authors on gastrointestinal microbiome and neuroscience research (T ≥ 8).

A total of 11,617 authors were involved in gastrointestinal microbiome research in the field of neuroscience. Cryan JF published the most papers (*n* = 100), followed by Dinan TG (*n* = 79), Clarke G (*n* = 38), Stanton C (*n* = 23), and O’Mahony SM (*n* = 19) (Table 3). The authors (*n* = 52) who published at least eight papers (T ≥ 8) belong to the core authors in this field and they were included to build the network map of core authors (Figure 4). The same color represented the same cluster.

**TABLE 3:** Top 10 productive and most co-cited authors on gastrointestinal microbiome and neuroscience research.

Rank	Author	Counts	%	Centrality	Institution	Country	Co-cited authors	Citations	TLS	Country
1	Cryan JF	100	4.40%	0.06	University College Cork	Ireland	Cryan JF	860	15,583	Ireland
2	Dinan TG	79	3.47%	0.06	University College Cork	Ireland	Dinan TG	632	14,926	Ireland
3	Clarke G	38	1.67%	0.13	University College Cork	Ireland	Bercik P	563	13,970	Canada
4	Stanton C	23	1.01%	0.01	University College Cork	Ireland	Desbonnet L	562	15,163	Ireland
5	O’Mahony SM	19	0.84%	0.01	University College Cork	Ireland	Mayer EA	459	10,349	USA
6	Bienenstock J	16	0.70%	0.07	McMaster University	Canada	O’Mahony SM	437	11,349	Ireland
7	Hashimoto K	15	0.66%	0.01	Chiba University	Japan	Bravo JA	428	9,476	Ireland
8	Burnet PWJ	14	0.62%	0	University of Oxford	England	Sampson TR	421	6,871	USA
9	Maes M	14	0.62%	0.02	Chulalongkorn University	Thailand	Maes M	416	6,872	Thailand
10	Neufeld KAM	13	0.57%	0.01	McMaster University	Ireland	Clarke G	408	10,881	Ireland

**FIGURE 5:**
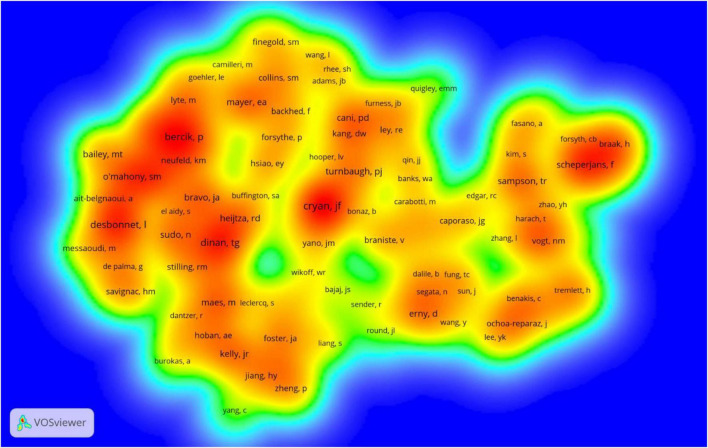
The density map of co-cited authors on gastrointestinal microbiome and neuroscience research (≥100).

Co-cited authors are authors who have been co-cited together in a range of publications (42). Among 75,329 co-cited authors, 119 were co-cited over 100. Cryan JF (*n* = 860) ranked first, followed by Dinan TG (*n* = 632), Bercik P (*n* = 563), Desbonnet L (*n* = 562), Mayer EA (*n* = 459). The remaining five top authors were co-cited from 408 to 437 (Table 3). The distribution of countries shows that the authors with high co-citation frequencies are mainly from Ireland and the United States. The authors (*n* = 119) with co-citations of at least 100 (T ≥ 100) were used to make the density map (Figure 5), this type of knowledge map could present the high-frequency co-cited authors clearly.

### Top active journals

To discover the most active journal research in gastrointestinal microbiome and neuroscience, we conducted citation journal analyses using VOSviewer. The results showed that 2,275 papers were published in 214 academic journals. Neurogastroenterology and Motility published the most papers (158, 6.95%), followed by Brain Behavior and Immunity (147, 6.46%), Frontiers in Neuroscience (127, 5.58%), Frontiers in Aging Neuroscience (72, 3.16%), and Journal of Alzheimer’s Disease (67, 2.95%) (Table 4). Among the top10 journals, five had an Impact Factor (IF) of more than five, and three were in the Q1 JCR division (Table 4).

**TABLE 4:** The top 10 journals publishing articles in the gastrointestinal microbiome and neuroscience.

Rank	Journal	Counts	%	IF (2021)	JCR	Country
1	Neurogastroenterology and Motility	158	6.95%	3.96	Q2	UK
2	Brain Behavior and Immunity	147	6.46%	19.23	Q1	USA
3	Frontiers in Neuroscience	127	5.58%	5.15	Q2	Switzerland
4	Frontiers in Aging Neuroscience	72	3.16%	5.70	Q1	Switzerland
5	Journal of Alzheimer’s Disease	67	2.95%	4.16	Q2	Netherlands
6	Frontiers in Neurology	63	2.77%	4.09	Q2	Switzerland
7	Prog Neuro-Psychoph	48	2.11%	5.20	Q2	UK
8	Behavioural Brain Research	47	2.07%	3.35	Q2	Netherlands
9	Neuroscience and Biobehavioral Reviews	43	1.89%	9.05	Q1	UK
10	Nutritional Neuroscience	43	1.89%	4.06	Q2	UK

### Top cited publications and co-cited references

Tables 5, 6 display the top 10 gastrointestinal microbiome-related cited articles and co-cited references in the discipline of neuroscience. The most often cited article on research in gastrointestinal microbiome and neuroscience was Cryan JF’s 2012 article on the impact of gut microbiota on the brain and behavior (Table 5). Four of the documents appeared on both lists, which were, respectively completed by the team of Cryan JF, Erny D, Sudo N, and Clarke G (Sudo et al., 2004; Cryan and Dinan, 2012; Clarke et al., 2013; Erny et al., 2015). The statistics imply that the articles on research in gastrointestinal microbiome and neuroscience by Cryan JF, Erny D, Sudo N, and Clarke G have been a significant contributor to the growth of the subject.

**TABLE 5:** Top 10 highly cited publications on research in gastrointestinal microbiome and neuroscience.

Rank	First author	Source (IF)	Publication year	Cited by	Document type
1	Cryan JF (Cryan and Dinan, 2012)	Nature Reviews Neuroscience (38.755)	2012	2,184	Review
2	Erny D (Erny et al., 2015)	Nature Neuroscience (28.771)	2015	1,427	Article
3	Sudo N (Sudo et al., 2004)	Journal of Physiology-London (6.229)	2004	1,405	Article
4	Foster JA (Foster and Neufeld, 2013)	Trends in Neurosciences (16.979)	2013	1,170	Review
5	Jiang HY (Jiang et al., 2015)	Brain Behavior and Immunity (19.227)	2015	984	Article
6	Clarke G (Clarke et al., 2013)	Molecular Psychiatry (13.437)	2013	967	Article
7	O’Mahony SM (O’Mahony et al., 2015)	Behavioural Brain Research (3.352)	2015	853	Review
8	Zheng P (Zheng et al., 2016)	Molecular Psychiatry (13.437)	2016	849	Article
9	Neufeld KM (Neufeld et al., 2011)	Neurogastroenterology and Motility (3.960)	2011	809	Article
10	Fung TC (Fung et al., 2017)	Nature Neuroscience (28.771)	2017	776	Review

**TABLE 6:** Top 10 highly co-cited references on research in gastrointestinal microbiome and neuroscience.

Rank	First author	Source (IF)	Publication year	Citations	Document type
1	Cryan JF (Cryan and Dinan, 2012)	Nature Reviews Neuroscience (38.755)	2012	396	Review
2	Bravo JA (Bravo et al., 2011)	P Natl Acad Sci USA (12.778)	2011	377	Article
3	Heijtz RD (Diaz et al., 2011)	P Natl Acad Sci USA (12.778)	2011	341	Article
4	Sudo N (Sudo et al., 2004)	Journal of Physiology-London (6.229)	2004	319	Article
5	Erny D (Erny et al., 2015)	Nature Neuroscience (28.771)	2015	299	Article
6	Sampson TR (Sampson et al., 2016)	Cell (66.849)	2016	286	Article
7	Clarke G (Clarke et al., 2013)	Molecular Psychiatry (13.437)	2013	244	Article
8	Hsiao EY (Hsiao et al., 2013)	Cell (66.849)	2013	243	Article
9	Scheperjans F (Scheperjans et al., 2015a)	Movement Disorders (9.698)	2015	243	Article
10	Bercik P (Bercik et al., 2011)	Gastroenterology (33.883)	2011	234	Article

### Analysis of keywords

**FIGURE 6:**
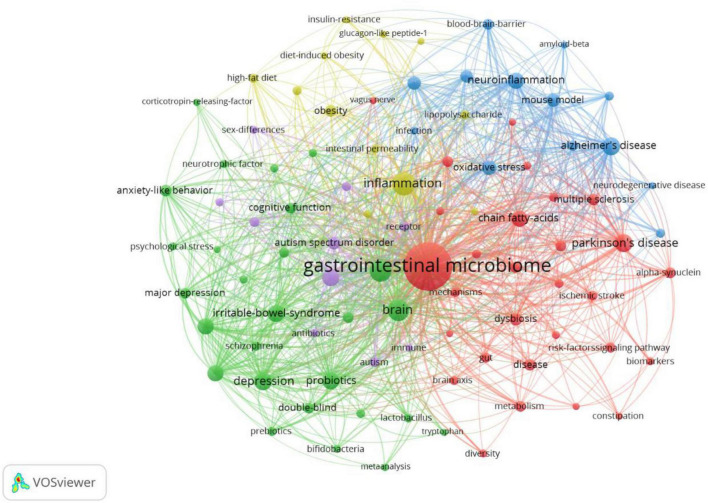
Co-occurrence and clustering of keywords in title/abstract fields of publications related to gastrointestinal microbiome and neuroscience. Map of keywords clustering showed 91 keywords with a minimum of 40 occurrences and divided into five clusters. Notes: The size of node and word reflects the co-occurrence frequencies, the link indicates the co-occurrence relationship, and nodes with the same color denote the same cluster.

A map of keyword co-occurrence reflects research hotspots. Co-occurrence and cluster analysis keywords were presented using VOSviewer (c). A total of 7,900 terms were extracted from keywords of all the 2,275 publications. High-frequency keywords (more than 40 times) were the subject of a clustering study. As shown in Figure 6, there were 91 nodes and 3,396 links in the network map, the size of each node indicates the occurrence of the keyword. As we can see from Table 7, the gastrointestinal microbiome was the highest frequency term with 1,850 co-occurrences, followed by inflammation, gut-brain axis, Parkinson’s disease, and Alzheimer’s disease. Five clusters were shown in different colors, and nodes with common attributes were classified into a color-coded cluster, represented by red, green, blue, yellow, and purple.

**TABLE 7:** The top 40 keywords related to research in gastrointestinal microbiome and neuroscience.

Rank	Keyword	Counts	Rank	Keyword	Counts
1	Gastrointestinal microbiome	1,850	21	Obesity	114
2	Inflammation	432	22	multiple sclerosis	109
3	Gut-brain axis	369	23	Cognitive function	108
4	Parkinson’s disease	278	24	Double-blind	104
5	Alzheimer’s disease	267	25	Major depression	104
6	Depression	265	26	Microbiota-gut-brain axis	99
7	Probiotics	245	27	Alpha-synuclein	98
8	Mice	230	28	Scfas	97
9	Irritable-bowel-syndrome	226	29	Metabolism	95
10	Chain fatty-acids	220	30	Diet	94
11	Stress	219	31	Blood-brain-barrier	91
12	Neuroinflammation	217	32	Cognitive impairment	91
13	Anxiety	198	33	Butyrate	90
14	Oxidative stress	166	34	Autism	84
15	Mouse model	154	35	Enteric nervous system	80
16	Central-nervous-system	143	36	Ischemic stroke	77
17	Dysbiosis	131	37	Mechanisms	77
18	Microglia	130	38	Risk-factors signaling pathway	77
19	Autism spectrum disorder	128	39	Animal-models	76
20	Anxiety-like behavior	116	40	Cytokines	76

**FIGURE 7:**
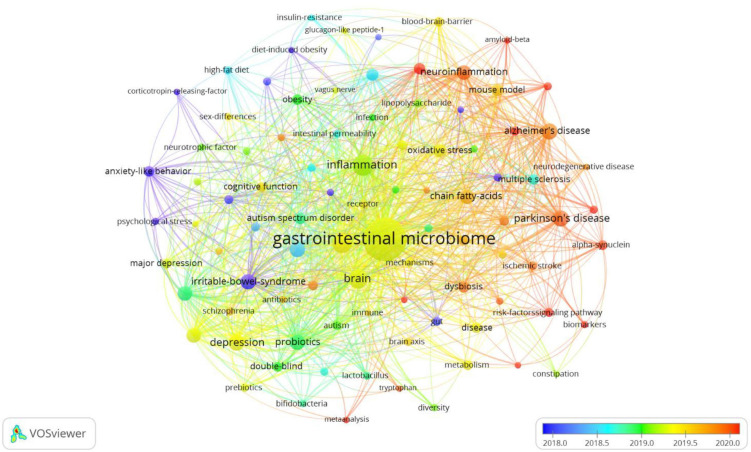
The overlay visualization map of keywords in title/abstract fields of publications related to gastrointestinal microbiome and neuroscience. Different colors were applied for each keyword based on their average appearance time in the overlay visualization map. Blue color represented the keywords that appeared relatively earlier than those in yellow and red upon time course.

In Figure 7, VOSviewer could mark keywords included in the overlay visualization map with different colors based on their average appearance year. The color blue stood for the keywords that appeared on the time course far earlier than those in yellow and red. As can be seen from Figure 7, large numbers of research hotspots related to gastrointestinal microbiomes and neuroscience have emerged in recent years, which indicates that the field is evolving at a tremendously fast pace.

### Analysis of bursts

#### Top 35 keywords with the strongest citation bursts

**FIGURE 8:**
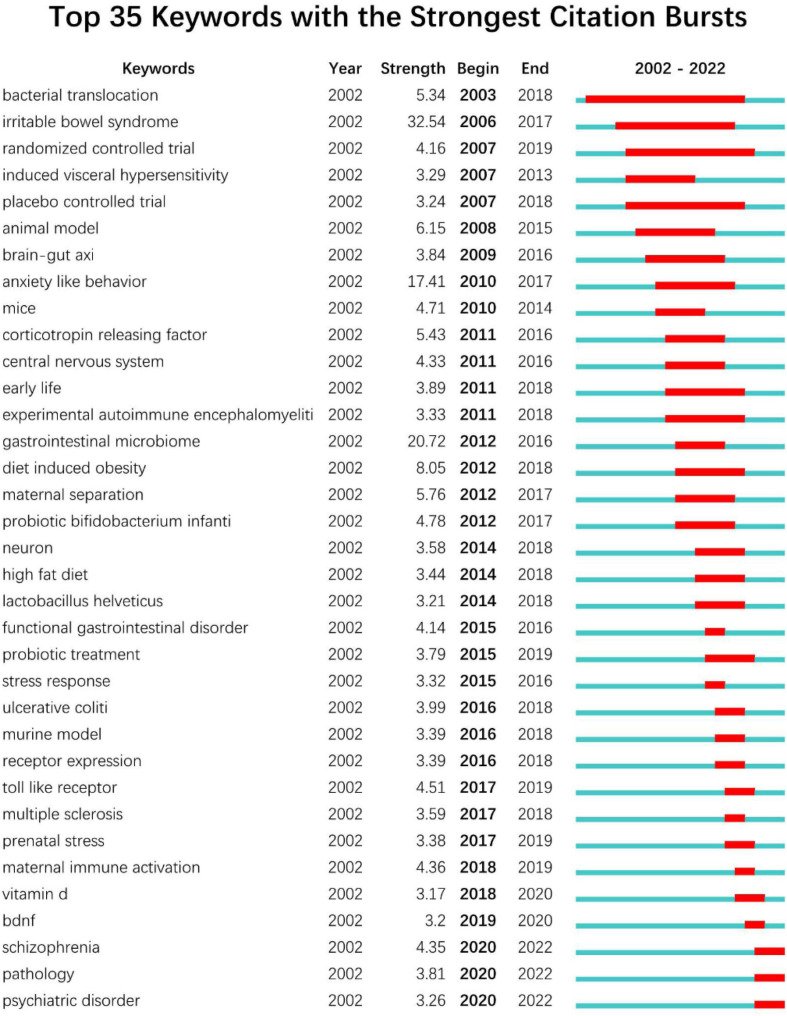
Top 35 Keywords with the Strongest Citation Bursts (sorted by the beginning year of burst) of the research in gastrointestinal microbiome and neuroscience during 2002–2022.

Burstiness of Keywords CiteSpace was employed to carry out burst keyword detection (Figure 8). Keyword burstiness can represent new academic trends, foretell future avenues for frontier study, and highlight prospective hotspots in a discipline. The burst detection is represented as a red segment on the blue timeline, which denotes the start year, end year, and duration of the burst. The timeline is shown as a blue line. We were especially interested in terms that have research relevance among the top 35 keywords with the highest burst intensity. These terms represented the research trends in the fields of gut microbiome and neuroscience (Figure 8). The duration of “bacterial translocation” outbreaks is the longest (2003–2018). Irritable bowel syndrome had the highest burst intensity from 2002 to 2022 (32.54), followed by gastrointestinal microbiome (20.72) and anxiety-like behavior (17.41). The top 35 keyword outbreak cycles with the most number of occurrences covered the entire period from 2002 to 2022. Notably, the burst of schizophrenia, pathology, and psychiatric disorder is still ongoing.

## Discussion

This bibliometric article analyzes publications research in gastrointestinal microbiome and neuroscience using visual analysis software. 2,275 publications from the WoSCC database in total were analyzed. The first stage (2002–2009) was the flat period of the development of this field, with less than 10 papers published annually. The second stage (2010–2015) was a slow growth period, the number of related publications showed an overall increasing annual trend, the number of published articles increased by dozens per year, and the annual number of published papers was less than 100. The third stage (2016–2022) was the rapid development stage, and the annual number of published papers was more than 100, accounting for more than 91% of the total number of included studies. The volume of literature will continue to grow in the future, and the research in this field will still attract the attention of scholars in the next few years.

### Knowledge structure of global

#### Countries/Regions

The United States was the leading contributor in this field with 732 publications, followed by China, Canada, Ireland, and England, which accounted for more than 73% of the total number of articles included in the study. GDP is an important factor in the number of publications of a country. The output of publications in the United States and China is far ahead, which is closely related to the top ranking of GDP (Figure 2A). One could presume that significant financial investments, such as the Human Microbiome Project (HMP) started by the NIH in 2007 and a remarkable research project on the gut microbiota-brain axis in 2013, affected research output on gut microbiota and neuroscience (Turnbaugh et al., 2007; Proctor, 2011; Human Microbiome Project Consortium, 2012, Robles-Alonso and Guarner, 2014). In addition, numerous experts in microbiology and neuroscience from Ireland, China, the United States, and other countries have studied the characteristics of the gastrointestinal microbiota in health and neuroscience disease states, the connection between pathological function and neuroscience disease, and other topics. These professionals, who serve as a foundation for the creation and publication of papers in this area, include Cryan JF, Dinan TG, Clarke G, Stanton C, O’Mahony SM, Bastiaanssen TFS, Yang C, Xie P, Bailey MT, Green SJ, Keshavarzian A, and Forsyth CB (Clarke et al., 2013; Dinan et al., 2014; Mayer et al., 2014; Stilling et al., 2015; Tarr et al., 2015; Dodiya et al., 2020; Joers et al., 2020; Li et al., 2020; van de Wouw et al., 2020; Frausto et al., 2021; Zheng et al., 2021; Connell et al., 2022; Teng et al., 2022). It is worth noting that the citations of articles published in the United States rank first, followed by Ireland and China. To some extent, the number of citations is related to the quality of the literature, but also to paying service, the time of publication of the paper, and the research hotspots or trend changes. In addition, it can be seen from the cooperation Network of countries/regions (Figure 2B) that compared with high-output countries such as the United States, England, and Australia, China has less cooperation with other countries in this field. To improve China’s influence in this field, it is necessary to improve the quality of literature and strengthen the exchange and cooperation with other high-output countries in this field.

### Institutions

A total of 2,618 institutions around the world have been studying the gastrointestinal microbiome in neuroscience. We can see from Table 2, University College Cork was the most productive and influential institution located in Ireland, and the top 10 institutions active in research of gastrointestinal microbiome and neuroscience came from the top 10 nations or regions. The top 10 institutions in this sector helped the United States, Australia, China, Ireland, and Canada overtake other countries as the most active ones in this field. Notably, as seen in Figure 3, whereas some of these institutions have worked closely together, some have not. Therefore, it is strongly advised that nations and institutions with similar research topics broaden and deepen their cooperation and work together to advance the development and prosperity of this field.

### Authors

Our results demonstrated that Cryan JF was the most productive author and the highest number of co-cited authors, who reviews the field of gut microbiota and neuroscience and discusses many theories (O’Mahony et al., 2009; Cryan and Dinan, 2012; Dinan and Cryan, 2012; Clarke et al., 2013; Mayer et al., 2014). The team represented by Cryan JF is active in this field and has more research results on the relationship between gastrointestinal microbiota and neuroscience, which plays an important guiding role. We also noticed that the top three frequently productive authors (Cryan JF, Dinan TG, and Clarke G) are all from the University College Cork (Table 3) and had a high centrality, suggesting that the University College Cork is an important institution in the research field. The co-occurrence map of authors (Figure 4) manifested there were active collaborations among authors in the same cluster, such as Cryan JF, Dinan TG, and O’Mahony SM, Clarke G, and Maes M, etc. Close cooperation was also observed among clusters, such as Dinan TG and Clarke G, Dinan TG and Neufeld KAM, Clarke G and Neufeld KAM, etc. However, some active authors on gastrointestinal microbiomes and neuroscience still lack collaboration with other scholars, such as the authors Hsiao EY, Bailey MT, and Han Y, etc., who have not yet formed a stable collaborative team.

### Journals

Most relevant studies were published in the journals (Q1/Q2 journals) with world-class influence, such as Brain Behavior and Immunity, Frontiers in Aging Neuroscience, and Neuroscience and Biobehavioral Reviews (Table 4). These results suggested that the link between gastrointestinal microbiota and neuroscience has attracted the attention of numerous scholars, and its research difficulties and value have also been recognized by scholars. All the top 10 journals are in the discipline of neuroscience and are established in the UK, Switzerland, Netherlands, and USA. When researchers produce and read studies on gastrointestinal microbiota and neuroscience, the top 10 journals may be given preference.

### Publications and references

The publications and references analysis has revealed the important references in the field of gastrointestinal microbiota and neuroscience in the past 20 years. The cited publications and co-cited references listed in Tables 5, 6 would provide an important reference for the study in this field. The findings revealed that multi-seed subjects closely related to research hotspots were highlighted in the top 10 highly cited publications and co-cited references. Four articles appear together in both lists (Tables 5, 6). The article with the most citations was on the impact of the gut microbiota on the brain and behavior by Cryan and Dinan (2012), which was published in Nature Reviews Neuroscience in 2012 and has a total of 2,184 citations. This research examines how the gut microbiota interacts with the central nervous system (CNS) *via* neural, endocrine, and immunological pathways, possibly affecting brain activity and behavior and influencing the regulation of anxiety, mood, cognition, and pain. The second article was published by Erny et al. (2015) from Nature Neuroscience in 2015. This study showed that the diverse microbiota partly restores microglia impairment while the host bacteria play a critical role in regulating microglia maturation and function. Research by Sudo et al. (2004) from the Journal of Physiology-London is the third most cited article. According to this study, the postnatal development of the hypothalamic-pituitary-adrenal (HPA) stress response in mice can be influenced by commensal bacteria. The fourth article came from Molecular Psychiatry and was produced by Clarke et al. (2013). This study showed that the absence of normal gut microbiota might seriously disturb 5-hydroxytryptamine neurotransmission in the central nervous system (CNS). The top 10 frequently cited publications and co-cited references may be given priority when researchers read and refer to articles on the gastrointestinal microbiome and neuroscience.

### Research focus and trends of global

According to the co-occurring keyword analysis, we identified some of the most important hotspots in this field over the past two decades, including the gastrointestinal microbiome, inflammation, gut-brain axis, Parkinson’s disease, and Alzheimer’s disease. The most widely studied diseases in this field are Parkinson’s disease and Alzheimer’s disease. Reichmann (2011) speculates that bacteria may be responsible for the development of PD, it may spread out from the enteric nervous system of the gut *via* the vagal nerve up to the brain. Since 2015, “Parkinso’s disease” has become the new top hotspot in this field. Gut microbiome composition is significantly associated with Parkinson’s disease (Scheperjans et al., 2015b). Kumari et al. (2020) studied the salivary metabolic profiling of saliva were studied in patients with PD (*n* = 76) and healthy controls (HC, *n* = 37) were analyzed and differentiated PD from HC. It is found that patients with PD might be characterized by metabolic imbalances like gut microflora system, energy metabolites, and neurotransmitters (Kumari et al., 2020). The ratio of Firmicutes to Bacteroidetes increased and bacterial diversity decreased in the gut of PD mice treated with rotenone (Yang et al., 2018). The abundance of Prevotella in PD patients is decreased, which affects the gastrointestinal dysfunction of PD patients (Mertsalmi et al., 2017). AD is the most common neurodegenerative disease. Priming of the innate immune system by the microbiota may enhance the inflammatory response to cerebral amyloids (such as amyloid-beta and alpha-synuclein), leading to neuro-system-related diseases such as Parkinson’s disease and Alzheimer’s disease (Friedland, 2015). Probiotic supplementation (Lactobacillus acidophilus, Lactobacillus bifidobacterium, and Lactobacillus fermentans) improves cognitive function and metabolic status in patients with Alzheimer’s disease (Akbari et al., 2016). Moreover, fecal microbiota transplant (FMT) technology, an ancient administration route traced back to fourth-century China (Zhang et al., 2012), is receiving increasing attention. Accumulating studies suggest that FMT has potential therapeutic effects on neuropsychiatric areas-related disorders, owing to the increase in microbiota diversity (Kang et al., 2021; Wang et al., 2021).

Cluster analysis is a statistical method for dividing the subjects of a particular field into several groups, which can reflect the research focus in the field (van Eck and Waltman, 2010; Xu et al., 2022). Our clustering analysis of keywords identified five focus areas of the research in gastrointestinal microbiome and neuroscience utilizing VOSviewer. Cluster 1 (red nodes) was the larger cluster with 29 co-occurrence terms: gastrointestinal microbiome, Parkinson’s disease, chain fatty acids, multiple sclerosis, short-chain fatty acids, alpha-synuclein, metabolism, ischemic stroke, mechanisms, enteric nervous system, nervous system, brain axis, metabolites, fecal microbiota transplantation, metabolomics, vagus nerve, etc. The topic of Cluster 1 is the mechanism of the gastrointestinal microbiome in Parkinson’s disease, multiple sclerosis, and ischemic stroke. Cluster 2 (green nodes) is primarily concerned with the effect of probiotics (e.g., lactobacillus, bifidobacteria) on depression, anxiety, major depression, schizophrenia, etc., and the “gut-brain axis” and “microbiota-gut-brain axis” are two important theories that are involved. This cluster includes 27 terms, such as gut-brain axis, depression, probiotics, anxiety, anxiety-like behavior, major depression, cognitive function, double-blind, microbiota-gut-brain axis, lactobacillus, schizophrenia, neurotrophic factor, bifidobacteria, psychological stress, etc. Cluster 3 (blue nodes) focuses on the mechanism of neurodegenerative diseases, such as Alzheimer’s disease and dementia, which contains 13 terms: Alzheimer’s disease, neuroinflammation, oxidative stress, mouse model, central-nervous-system, microglia, blood-brain-barrier, cognitive impairment, dementia, neurodegenerative disease, amyloid-beta, etc. Cluster 4 (yellow nodes) is mainly related to “gastrointestinal microbiome, inflammation: a link between obesity, insulin resistance, and cognition” with 12 terms: inflammation, obesity, high-fat diet, intestinal permeability, lipopolysaccharide, TNF-alpha, bacterial translocation, insulin-resistance, glucagon-like peptide-1, etc. The topic of Cluster 5 (purple nodes), which includes the keywords mice, autism spectrum disorder, autism, animal models, immune system, sex differences, and more, is “the function of the gut microbiome in autism spectrum disorder and autism.” Despite the increasing recognition of these hotspots, as these research directions develop, further innovations and breakthroughs may be hindered. Therefore, in the coming years, it will be important to focus more on the frontier research topics that the burst keyword analysis has shown.

The “burst keywords” can be categorized into three phases based on when they started and ended. Studies in this field initially concentrated on controlled trials involving bacterial translocation and irritable bowel syndrome from 2003 to 2007 (O’Mahony et al., 2009; Dinan and Cryan, 2012; Kennedy et al., 2012; Park et al., 2013; Garate et al., 2014; Brzozowski et al., 2016; Pigrau et al., 2016; Azpiroz et al., 2017; Morris et al., 2017; Sundman et al., 2017; Lin et al., 2018; Roomruangwong et al., 2018, 2019). The second stage, which lasted from 2008 to 2014, concentrated on animal trials involving anxiety, the central nervous system, diet-induced obesity, experimental autoimmune encephalomyelitis, and the brain-gut axis (Ochoa-Reparaz et al., 2011; Foster and Neufeld, 2013; Winek et al., 2016; Scott et al., 2017; Vaughn et al., 2017). The research flora focused on lactobacillus helveticus and probiotic bifidobacterium, while the modeling method concentrated on the high-fat diet (McKernan et al., 2010; Ohland et al., 2013; Ait-Belgnaoui et al., 2014; Kang et al., 2014; Mayer et al., 2014; Liang et al., 2015; Magnusson et al., 2015; Guillemot-Legris et al., 2016; Baker et al., 2017; Li et al., 2018; Schachter et al., 2018; Hassan et al., 2019; Beraldi et al., 2020). After 2015, the main diseases studied were functional gastrointestinal disorders, stress response, ulcerative colitis, prenatal stress, schizophrenia, and mental disorders (Daulatzai, 2015; Schmidt et al., 2015; Davis et al., 2016; Evrensel and Ceylan, 2016; Gur et al., 2017, 2019; Abautret-Daly et al., 2018; Jasarevic et al., 2018; Ju et al., 2020; Bioque et al., 2021; Luo et al., 2021; Yanguas-Casas et al., 2021; Urbonaite et al., 2022). Brain-derived neurotrophic factors and Toll-like receptors were the key research factors (Zeraati et al., 2019; Haghighat et al., 2021). Pathological experiments were the primary study methodology, while probiotic therapy was the primary research topic (Akbari et al., 2016; Kozyrev et al., 2020; Bannerman et al., 2022; Pochakom et al., 2022). Notably, including “schizophrenia” (burst strength 4.35), “pathology” (burst strength 3.81), and “psychiatric disorder” (burst strength 3.26), which are the current research frontiers in this field and are currently within the burst period.

## Conclusion

In this study, 2,275 original studies from 2002 to 2022 (August 20, 2022) related to the research in gastrointestinal microbiome and neuroscience were downloaded from the WoSCC database and analyzed using VOSviewer to generate knowledge maps. The number of articles on the research in gastrointestinal microbiome and neuroscience has increased rapidly in recent years. The United States and China have made the most notable contributions to the research in gastrointestinal microbiomes and neuroscience. Research on “gastrointestinal microbiome, inflammation: the link between obesity, insulin-resistance and cognition” and “the role of two important theories of the gut-brain axis and microbial-gut-brain axis in diseases in neuroscience” will continue to be the hotspot. Further studies on the mechanisms linking gastrointestinal microbiome and neuroscience will promote neuroscience disease therapy.

## Data availability statement

The original contributions presented in this study are included in the article/supplementary material, further inquiries can be directed to the corresponding author.

## Author contributions

DL and YD conceived the study and performed critical revision of the manuscript. JY designed the study, performed statistical analyses, and drafted the manuscript. YC, YL, LP, and ZL performed the article retrieval, data interpretation, and provided supervision. All authors read and approved the final manuscript for publication.

## Footnotes

1
https://www.webofscience.com/wos/woscc/summary/156f3a2d-3623-4356-9a8a-55d8dbeeecc8-56b9ffef/relevance/1

2
https://sourceforge.net/
